# Differences in complications between hepatitis B-related cirrhosis and alcohol-related cirrhosis

**DOI:** 10.1515/med-2021-0401

**Published:** 2021-12-06

**Authors:** Yu-Pei Zhuang, Si-Qi Wang, Zhao-Yu Pan, Hao-Jie Zhong, Xing-Xiang He

**Affiliations:** Department of Gastroenterology, The First Affiliated Hospital of Guangdong Pharmaceutical University, No. 19 Nonglinxialu, Guangzhou 510000, Guangdong Province, China

**Keywords:** alcoholism, hepatitis B virus, liver cirrhosis, patients

## Abstract

**Objectives:**

This study aimed to investigate the differences in complications between hepatitis B virus (HBV)-related and alcohol-related cirrhoses.

**Methods:**

Medical records of patients with HBV-related and alcohol-related cirrhoses treated from January 2014 to January 2021 were, retrospectively, reviewed. The unadjusted rate and adjusted risk of cirrhotic complications between the two groups were assessed.

**Results:**

The rates of hepatocellular carcinoma (HCC) and hypersplenism were higher in HBV-related cirrhosis (both *P* < 0.05), whereas the rates of hepatic encephalopathy (HE) and acute-on-chronic liver failure (ACLF) were higher in alcohol-related cirrhosis (both *P* < 0.05). After adjusting for potential confounders, HBV-related cirrhotic patients had higher risks of HCC (odds ratio [OR] = 34.06, 95% confidence interval [CI]: 4.61–251.77, *P* = 0.001) and hypersplenism (OR = 2.29, 95% CI: 1.18–4.42, *P* = 0.014), whereas alcohol-related cirrhotic patients had higher risks of HE (OR = 0.22, 95% CI: 0.06–0.73, *P* = 0.013) and ACLF (OR = 0.30, 95% CI: 0.14–0.73, *P* = 0.020).

**Conclusion:**

Cirrhotic patients with different etiologies had different types of complications: HBV-related cirrhotic patients exhibited increased risks of HCC and hypersplenism and alcohol-related cirrhotic patients more readily developing HE and ACLF.

## 1 Introduction

Cirrhosis, as an advanced stage of chronic liver disease, and its complications are associated with high morbidity and cause more than 1 million deaths worldwide annually [1]. Chronic infection with hepatitis B virus (HBV) and alcoholism are the main etiologies [1]. In 2015, nearly 240 million people globally had HBV infection, and there were approximately 0.45 million deaths due to HBV-related cirrhosis and its complications [2]. Additionally, the rate of alcohol-related cirrhosis is growing with the rapid increase in alcohol consumption [3]. In 2010, nearly 0.5 million deaths worldwide were caused by alcohol-related cirrhosis, which accounted for approximately 50% of all cirrhosis-related deaths [4].

Cirrhotic patients frequently develop complications, and those with complications had worse outcomes (including higher mortality) than those without complications [5,6]. For example, cirrhotic patients with severe hepatic encephalopathy (HE) had a first-year mortality rate of more than 50% [7,8]. Additionally, cirrhotic patients with acute-on-chronic liver failure (ACLF) had a high 28 day mortality rate caused by acute decompensation, organ failure, and/or serious systemic inflammation [9]. Furthermore, hepatocellular carcinoma (HCC), as a major cause of cancer-associated death, has a very poor prognosis and a 5 year survival rate of less than 15% [10].

Cirrhosis cases with different etiologies present with different clinical characteristics [11,12]. It is very important to identify the differences in cirrhosis-related complications between different etiologies, which may affect prognosis, to guide treatment planning and thereby improve prognosis. Thus, this study aimed to distinguish the cirrhotic complications between patients with HBV-related and alcohol-related cirrhoses.

## 2 Methods

### 2.1 Study design

The electronic medical data of hospitalized patients with alcohol-related and HBV-related cirrhoses, from January 2014 to January 2021, were reviewed, retrospectively. The exclusion criteria were as follows: (1) other underlying liver diseases (such as autoimmune liver disease), other viral-related cirrhosis, Wilson’s disease, primary biliary cirrhosis, concomitant alcohol abuse, and HBV infection; (2) carcinoma (excluding HCC); and (3) severe cardiac or pulmonary diseases. The sample size of patients was estimated using an online software (Power and Sample Size Calculators; HyLown Consulting LLC, Atlanta, GA, USA).

The research related to human use has been complied with all the relevant national regulations, institutional policies, and in accordance with the tenets of the Helsinki Declaration and has been approved by the ethics review committee of the First Affiliated Hospital of Guangdong Pharmaceutical University (ethics review number: 202110). The need for the patient informed consent was waived as retrospective anonymized data were used in this study.

### 2.2 Data collection

Patient medical data were extracted from the hospital information system as follows: demographics, alcoholism, smoking status, medical history (including cirrhosis etiology, diabetes, and hypertension), clinical presentation, imaging results, and laboratory results including alanine transaminase (ALT), aspartate transaminase (AST), bilirubin, serum albumin, lipid profile, international normalized ratio (INR), prothrombin time (PT), routine examination of blood, and serum ammonia.

### 2.3 Definitions

The cirrhosis diagnosis was based on pathological findings or a combination of clinical presentation and imaging and laboratory results [13]. Chronic HBV infection was defined based on serum hepatitis B surface antigen positivity for >6 months. Alcohol-related cirrhosis was defined as cirrhosis together with alcoholism (alcohol consumption ≥20 g/day in women and ≥40 g/day in men for >5 years) in the absence of other liver diseases [14]. ACLF was defined as INR ≥1.5 and serum bilirubin ≥5 mg/dL, complicated by encephalopathy and/or ascites within 4 weeks, in cirrhotic patients [15]. HE was defined as abnormal neuropsychiatric manifestations and an abnormal ammonia level [16]. Hypersplenism was defined as imaging results suggesting splenomegaly and platelet count <120 × 10^9^/L [17] or a history of splenectomy owing to hypersplenism. Leukopenia, thrombocytopenia, and erythropenia were diagnosed based on white blood cell count <4.0 × 10^9^/L, platelet count <100 × 10^9^/L, and red blood cell count <3.5 × 10^12^/L for women or <4.0 × 10^12^/L for men, respectively. Smoking was defined as a history of smoking for more than 1 year. Moreover, the duration of liver disease was estimated using the age at alcoholism or HBV infection onset.

### 2.4 Statistical analyses

Categorical variables were described as frequency (percentage) and were analyzed with chi-square tests. Normally distributed continuous variables are described as mean ± standard deviation and were analyzed with unpaired two-tailed Student’s *t*-tests, whereas other continuous variables are described as median (interquartile range) and were analyzed with Mann–Whitney *U* tests. Logistic regression with backward stepwise selection was used to determine the risks of complications by cirrhosis etiology, adjusting for sex, age, body mass index, hypertension, diabetes, disease duration, smoking, bilirubin, and albumin. The results are described as odds ratios (ORs) with 95% confidence intervals (CIs). *P* values less than 0.05 (two-tailed) indicated statistical significance. SPSS statistical software (version 22; IBM Corporation, Armonk, NY, USA) was used for all statistical analyses.

## 3 Results

### 3.1 Patient characteristics

We, retrospectively, enrolled a total of 514 cirrhotic patients, comprising 445 with HBV-related cirrhosis and 69 with alcohol-related cirrhosis. Table 1 lists their demographic and clinical characteristics. Among the patients with HBV-related cirrhosis, 401 (90.11%) were taking antiviral therapy and 156 (60.94%) had a low HBV DNA level (defined as <2,000 IU/mL).

**Table 1:** Patient characteristics

	HBV-related cirrhosis (*n* = 445)	Alcohol-related cirrhosis (*n* = 69)	*P*-value
Age (years)	58.86 ± 13.53	58.65 ± 12.1	0.905
Sex (male)	368 (82.92)	69 (100)	<0.001
BMI (kg/m^2^)	22.45 (20.55–24.98) (*n* = 381)	21.55 (19.47–25.01) (*n* = 51)	0.126
Disease duration (years)	*n* = 298	*n* = 59	0.455
<10	89 (29.87)	20 (33.90)	
10–19	92 (30.87)	21 (35.59)	
≥20	117 (39.26)	18 (30.51)	
Diabetes	90 (20.22)	20 (28.99)	0.114
Hypertension	135 (30.34)	25 (36.23)	0.331
Smoking	130 (29.21)	52 (75.36)	<0.001

Data are presented as mean ± standard deviation, *n* (%), or median (interquartile range). BMI, body mass index and HBV, hepatitis B virus.

### 3.2 Differences in hepatic function indexes by cirrhosis etiology

HBV-related cirrhotic patients had a higher rate of Child–Pugh grades B and C (81.50 vs 18.50%, *P* = 0.008), and alcohol-related cirrhotic patients had a lower serum albumin level (32.28 ± 7.02 vs 34.41 ± 6.38 mmol/L, *P* = 0.017). However, no differences were found in the levels of AST, ALT, bilirubin, PT, INR, or blood lipids between the two groups (Table 2).

**Table 2:** Differences in hepatic function indexes by cirrhosis etiology

	HBV-related cirrhosis (*n* = 445)	Alcohol-related cirrhosis (*n* = 69)	*P*-value
AST (U/L)	30.00 (18.00–52.00) (*n* = 441)	25.00 (15.85–40.00) (*n* = 69)	0.152
ALT (U/L)	38.25 (26.00–82.75) (*n* = 441)	50.00 (29.00–100.00) (*n* = 69)	0.190
Bilirubin (g/L)	18.20 (12.00–33.55) (*n* = 441)	23.20 (12.80–55.60) (*n* = 69)	0.057
ALB (mmol/L)	34.41 ± 6.38 (*n* = 441)	32.28 ± 7.02 (*n* = 69)	0.017
TC (mmol/L)	3.83 (3.03–4.56) (*n* = 249)	3.84 (2.92–4.51) (*n* = 49)	0.980
TG (mmol/L)	0.97 (0.66–1.39) (*n* = 249)	1.02 (0.81–1.49) (*n* = 49)	0.131
HDL-C (mmol/L)	2.27 (1.69–2.80) (*n* = 249)	2.07 (1.48–3.09) (*n* = 49)	0.819
LDL-C (mmol/L)	1.02 (0.82–1.29) (*n* = 249)	1.02 (0.77–1.20) (*n* = 49)	0.362
PT (s)	14.70 (13.60–16.50) (*n* = 424)	14.75 (13.70–17.08) (*n* = 68)	0.755
INR	1.15 (1.05–1.34) (*n* = 424)	1.16 (1.05–1.40) (*n* = 68)	0.803
Child–Pugh classification	(*n* = 421)	(*n* = 68)	0.008
A	236 (56.06)	26 (38.24)	
B/C	185 (81.50)	42 (18.50)	

Data are presented as mean ± standard deviation, *n* (%), or median (interquartile range). ALB, albumin; ALT, alanine transaminase; AST, aspartate transaminase; HBV, hepatitis B virus; HDL-C, high-density lipoprotein cholesterol; INR, international normalized ratio; LDL-C, low-density lipoprotein cholesterol; PT, prothrombin time; TC, total cholesterol; and TG, triacylglycerol.

### 3.3 Differences in complications by cirrhosis etiology

The rates of HE and ACLF were higher in alcohol-related cirrhotic patients than HBV-related cirrhotic patients (HE: 15.94 vs 4.49%, *P* = 0.001; ALCF: 7.25 vs 2.28%, *P* = 0.040). The serum ammonia level was also higher in alcohol-related cirrhotic patients with HE than HBV-related cirrhotic patients with HE (134.00 ± 62.99 vs 82.16 ± 17.82 µmol/L, *P* = 0.029). In contrast, the rates of HCC and hypersplenism were higher in HBV-related cirrhotic patients than alcohol-related cirrhotic patients (HCC: 39.55 vs 1.45%, *P* < 0.001; hypersplenism: 45.84 vs 28.99%, *P* = 0.009). No significant differences in the rates of jaundice, ascites, esophageal and gastric varices, or spontaneous peritonitis were observed between the two groups (Table 3).

**Table 3:** Differences in complications by cirrhosis etiology

	HBV-related cirrhosis (*n* = 445)	Alcohol-related cirrhosis (*n* = 69)	*P*-value
Jaundice	32 (7.26) (*n* = 441)	9 (13.04)	0.148
Esophageal and gastric varices	213 (47.87)	35 (50.74)	0.699
Ascites	180 (40.45)	34 (49.28)	0.190
Hypersplenism	204 (45.84)	20 (28.99)	0.009
Spontaneous bacterial peritonitis	16 (3.60)	1 (1.45)	0.714
ACLF	10 (2.28) (*n* = 439)	5 (7.25)	0.040
HE	20 (4.49)	11 (15.94)	0.001
HCC	176 (39.55)	1 (1.45)	<0.001

Data are presented as *n* (%). ACLF, acute-on-chronic liver failure; HBV, hepatitis B virus; HCC, hepatocellular carcinoma; and HE, hepatic encephalopathy.

### 3.4 Adjusted risk of complications by cirrhosis etiology

Adjusted logistic regression indicated that the risks of HE (OR = 0.22, 95% CI: 0.06–0.73, *P* = 0.013) and ACLF (OR = 0.02, 95% CI: 0.14–0.73, *P* = 0.020) were higher in patients with alcohol-related cirrhosis than those with HBV-related cirrhosis (Table 4). In contrast, the risks of HCC (OR = 34.06, 95% CI: 4.61–251.77, *P* = 0.001) and hypersplenism (OR = 2.29, 95% CI: 1.18–4.42, *P* = 0.014) were higher in HBV-related cirrhotic patients than alcohol-related cirrhotic patients.

**Table 4:** Adjusted risks of complications by cirrhosis etiology

	OR	95% CI	*P*-value
Jaundice	—	—	—
Esophageal and gastric varices	—	—	—
Ascites	—	—	—
Hypersplenism	2.29	1.18–4.42	0.014
Spontaneous bacterial peritonitis	—	—	—
ACLF	0.30	0.14–0.73	0.020
HE	0.22	0.06–0.73	0.013
HCC	34.06	4.61–251.77	0.001

Adjusted for sex, age, body mass index, hypertension, diabetes, disease duration, smoking, bilirubin, and albumin. ACLF, acute-on-chronic liver failure; CI, confidence interval; HBV, hepatitis B virus; HCC, hepatocellular carcinoma; HE, hepatic encephalopathy; and OR, odds ratio.

### 3.5 Differences in cytopenia by cirrhosis etiology

The rate of leukopenia was notably higher, and the rate of erythropenia was lower in HBV-related cirrhotic patients than alcohol-related cirrhotic patients (leukopenia: 18.33 vs 7.26%, *P* = 0.034; erythropenia: 48.14 vs 64.18%, *P* = 0.018). No significant difference was observed in the rate of thrombocytopenia between the two groups (Table 5). After adjusting for confounders, the risk of erythropenia (OR = 0.13, 95% CI: 0.02–0.85, *P* = 0.033) was higher in alcohol-related cirrhotic patients, whereas the risk of leukopenia (OR = 13.39, 95% CI: 1.80–99.76, *P* = 0.011) was higher in HBV-related cirrhotic patients (Table 6).

**Table 5:** Differences in cytopenia by cirrhosis etiology

	HBV-related cirrhosis (*n* = 445)	Alcohol-related cirrhosis (*n* = 69)	*P*-value
Leukopenia	79 (18.33) (*n* = 431)	5 (7.46) (*n* = 67)	0.034
Erythropenia	207 (48.14) (*n* = 431)	43 (64.18) (*n* = 67)	0.018
Thrombocytopenia	158 (36.66) (*n* = 431)	31 (46.27) (*n* = 67)	0.138

Data are presented as *n* (%). HBV, hepatitis B virus.

**Table 6:** Adjusted risks of cytopenia by cirrhosis etiology

	OR	95% CI	*P*-value
Leukopenia	13.39	1.80–99.76	0.011
Erythropenia	0.13	0.02–0.85	0.033
Thrombocytopenia	—	—	—

Adjusted for sex, age, body mass index, hypertension, diabetes, disease duration, smoking, bilirubin, and albumin. CI, confidence interval; and OR, odds ratio.

## 4 Discussion

In the present study, the rates of complications between HBV-related and alcohol-related cirrhoses differed, with HBV-related cirrhotic patients having higher adjusted risks of HCC and hypersplenism than alcohol-related cirrhotic patients. In contrast, the adjusted risks of HE and ACLF were notably greater in alcohol-related cirrhotic patients, indicating that cirrhotic patients with different etiologies had different types of complications.

A retrospective cohort study indicated that the rate of HCC in HBV-related cirrhotic patients was higher than that in alcohol-related cirrhotic patients (32.6 vs 6.0%) [18]. Consistently, we observed that the rate of HCC was notably higher in HBV-related cirrhotic patients than alcohol-related cirrhotic patients (39.55 vs 1.45%). Another study revealed that viral hepatitis promoted the development of HCC more than alcoholic hepatitis, which meant that patients with HCC due to viral hepatitis had worse outcomes [19]. Therefore, rigorous HCC surveillance is needed in HBV-related cirrhotic patients.

We also found that HBV-related cirrhotic patients had a higher rate of hypersplenism than alcohol-related cirrhotic patients (45.84 vs 28.99%). As one of the most common cirrhotic complications, hypersplenism often causes rapid and premature destruction of blood cells, especially platelets and leukocytes, which can lead to infection and bleeding [20]. A study reported that leukopenia in cirrhotic patients led to a much higher risk of infection and poor prognosis [21]. We discovered that the rate of leukopenia was substantially higher in HBV-related cirrhotic patients than alcohol-related cirrhotic patients (18.33 vs 7.26%). Thus, more attention should be paid to preventing infection among patients with HBV-related cirrhosis. In contrast, erythropenia was more common in alcohol-related cirrhotic patients (64.18 vs 48.14%). This may be because alcohol suppresses erythropoiesis in the bone [22,23].

We found that the rate of HE was significantly higher in alcohol-related cirrhotic patients than HBV-related cirrhotic patients (15.94 vs 4.49%). Similarly, a retrospective cohort study of 598 cirrhotic patients by Vaz et al. [24] reported that alcohol-related cirrhotic patients had a higher rate of HE than hepatitis C virus-related cirrhotic patients (11.0 vs 5.0%). This may be because chronic alcohol abuse may cause more severe neocortical injury and cognition impairment than chronic hepatitis virus infection [25]. Furthermore, the severity of HE has been shown to be associated with the serum ammonia level [26], and we found that alcohol-related cirrhotic patients with HE had a higher serum ammonia level than HBV-related cirrhotic patients with HE (134.00 ± 62.99 vs 82.16 ± 17.82 µmol/L), indicating more severe HE in alcohol-related cirrhotic patients. Therefore, early detection and treatment of HE in alcohol-related cirrhotic patients should be considered.

Additionally, we found that the rate of ACLF was higher in alcohol-related cirrhotic patients than HBV-related cirrhotic patients (7.25 vs 2.28%). Our result was similar to that of research by Axley et al. [27], which demonstrated that the rate of ACLF was clearly higher in alcohol-related cirrhotic patients than hepatitis virus-related cirrhotic patients (7.2 vs 4.1%). Moreover, another study reported that alcohol-related cirrhotic patients exhibited more severe ACLF than patients with viral liver disease [28]. Thus, it is necessary to be aware of the high risk of ACLF when treating patients with alcohol-related cirrhosis.

There were, admittedly, several limitations in our study, particularly the small sample size. Additionally, we only included hospitalized patients (who may have had greater disease severity), which may have caused selection bias. Furthermore, several potential confounders, such as diet (especially a high-protein diet), that may influence the occurrence of HE in cirrhotic patients, were not considered. Future studies with large sample sizes are warranted to confirm our findings.

## 5 Conclusions

Cirrhotic patients with different etiologies had different types of complications, with HBV-related cirrhotic patients exhibiting increased risks of HCC and hypersplenism and alcohol-related cirrhotic patients more readily developing HE and ACLF. These findings provide evidence supporting the hypothesis that cirrhosis is not a single disease. There is a need for further classification to make better informed decisions related to complication prevention and treatment.
